# What you saw is what you will hear: Two new illusions with audiovisual postdictive effects

**DOI:** 10.1371/journal.pone.0204217

**Published:** 2018-10-03

**Authors:** Noelle R. B. Stiles, Monica Li, Carmel A. Levitan, Yukiyasu Kamitani, Shinsuke Shimojo

**Affiliations:** 1 Biology and Biological Engineering, California Institute of Technology, Pasadena, California, United States of America; 2 Cognitive Science, Occidental College, Los Angeles, California, United States of America; 3 Graduate School of Informatics, Kyoto University, Kyoto, Japan; 4 ATR Computational Neuroscience Laboratories, Seika, Soraku, Kyoto, Japan; Centre de neuroscience cognitive, FRANCE

## Abstract

Neuroscience investigations are most often focused on the prediction of future perception or decisions based on *prior* brain states or stimulus presentations. However, the brain can also process information retroactively, such that *later* stimuli impact conscious percepts of the stimuli that have already occurred (called “postdiction”). Postdictive effects have thus far been mostly unimodal (such as apparent motion), and the models for postdiction have accordingly been limited to early sensory regions of one modality. We have discovered two related multimodal illusions in which audition instigates postdictive changes in visual perception. In the first illusion (called the “Illusory Audiovisual Rabbit”), the location of an illusory flash is influenced by an auditory beep-flash pair that follows the perceived illusory flash. In the second illusion (called the “Invisible Audiovisual Rabbit”), a beep-flash pair following a real flash suppresses the perception of the earlier flash. Thus, we showed experimentally that these two effects are influenced significantly by postdiction. The audiovisual rabbit illusions indicate that postdiction can bridge the senses, uncovering a relatively-neglected yet critical type of neural processing underlying perceptual awareness. Furthermore, these two new illusions broaden the Double Flash Illusion, in which a single real flash is doubled by two sounds. Whereas the double flash indicated that audition can create an illusory flash, these rabbit illusions expand audition’s influence on vision to the suppression of a real flash and the relocation of an illusory flash. These new additions to auditory-visual interactions indicate a spatio-temporally fine-tuned coupling of the senses to generate perception.

## Introduction

Postdiction occurs when later sensory stimuli impact the perception of already-presented stimuli [1–3]. This type of perceptual processing is particularly prevalent in short time scales of less than a quarter of a second. Several known perceptual effects are postdictive in nature. For instance, apparent motion occurs when a stimulus is presented at two discrete locations but is perceived to move continuously and smoothly across the screen from the first to the second location [4–7]. This effect is, at least in part, postdictive as the smooth motion perception occurs even if the direction of the stimuli movement is randomized (and consequently cannot be predicted) [8]. The second flash’s location retroactively influences the perception of the motion trajectory, generating the impression of smooth movement between the two locations. Another example of postdiction, the cutaneous rabbit illusion, occurs when the forearm is tapped five times in three separate locations (2 ms per tap, separated by 40–80 ms) [9]. The taps are then perceived to be distributed (approximately uniformly) along the forearm rather than in 3 discrete locations [9]. Depending on the number of the taps, the position perceived for each tap is influenced retroactively so that the taps are evenly distributed between the locations of first and last tap. Other effects that are at least in part postdictive include backward masking, the flash lag effect, and the TMS-triggered scotoma effect [4, 5, 9–15]. To date, most postdictive effects that have been reported occur within one modality (one notable exception being the crossmodal flash-lag [10], which is audiovisual). Accordingly, models for how postdiction might be processed in the sensory regions of the brain have been primarily limited to the sensory regions of one modality. (Note: We will review the multisensory literature and the relevance of our experiments to previous effects in the discussion section.)

Models of the neural processing of postdiction explain how a stimulus presented later in time may impact a stimulus presented earlier [1, 15, 16]. For example, if a later-presented stimulus can “catch-up” to an earlier one in the processing pipeline, then the two stimuli can be integrated, and perception modified accordingly (Note: this type of model is based on the concept that conscious perception only occurs many milliseconds following stimuli presentation [15, 17]). Several other computational and neural models also exist and will be discussed in more depth in the discussion section.

**Fig 1:**
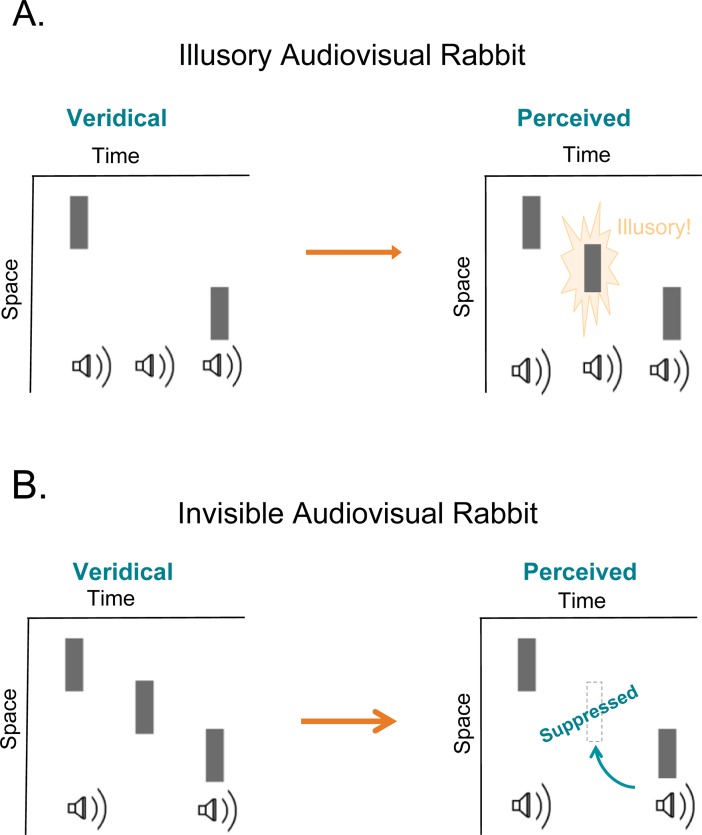
Space-time diagrams of the illusory and invisible AV Rabbits. Plots of space and time dimensions indicate the approximate timing and location of the flashes and beeps presented in the Illusory and Invisible AV Rabbits. The veridical plot indicates the physical stimulus presented to the participants, and the perceived plot indicates the stimulus that participants report seeing and hearing. Vertical bars represent visual flashes, and speaker icons represent sounds.

In this paper we present two new postdictive effects that use spatial motion (as in apparent motion and cutaneous rabbit effects), but also rely on the combination of visual and auditory perception. In the first illusion, the Illusory Audiovisual Rabbit, with a sequence of [beep-flash, beep, beep-flash], an illusory flash is perceived during the second beep (all beeps are presented in the same central location). The illusory flash location is determined by the location of the beep-flash pair presented after it (Fig 1A and S1 Movie; originally reported by Kamitani *et al*. in 2001 in abstract form). In the second illusion, the Invisible Audiovisual Rabbit, with a sequence of [beep-flash, flash, beep-flash], the second flash is suppressed by the presence of a flash-beep pair presented after it (Fig 1B and S2 Movie). These new illusions indicate that an illusory flash can be assigned a location postdictively, and that visual perception can be suppressed postdictively. Moreover, these new audiovisual effects indicate that crossmodal postdictive processing occurs, and broaden the types of crossmodal effects, which have postdictive processing. We will briefly explore modifications to the current postdictive neural models that could explain these two new crossmodal illusions.

## Materials and methods

### Overview

We will describe ten different experiments, though some experiments were conducted within the same experimental block and experimental session. (We chose this way of description for a more straightforward communication.) We will refer to an experimental block to be a set of trials with interleaved conditions all with the same questions. We will refer to a session as a time period in which multiple blocks were performed with one or more experiments in each block. Five of these experiments demonstrate the Illusory Audiovisual Rabbit (Experiments 1.1–1.5) and five of them demonstrate the Invisible Audiovisual Rabbit (Experiments 2.1–2.5). Experiments 1.1, and 2.1 were conducted in the same block, with participants reporting how many flashes they perceived when experiencing various combinations of beeps and flashes. Experiment 2.2 was designed to test if the Invisible AV rabbit is postdictive in nature, and required the reporting of the number of flashes perceived. Experiments 1.2 and 2.3 both asked participants to report the locations of the flashes that they perceived, but were conducted in separate blocks. Experiment 1.3 was designed to test whether the perceived location of an illusory flash is generated postdictively, by randomly varying the direction of movement between the physical flashes. Experiments 1.4 and 2.4 were conducted in the same block, and probed the strength of the illusions at different eccentricities. Experiments 1.5 and 2.5 were conducted in the same block, and asked participants to rate their confidence in their responses about the number of flashes that they perceived.

### Participants

Thirteen participants (4 male and 9 female) took part in the experiments, though some participants did not complete all experiments. Subjects were all naive and told only the task they needed to complete (*i*.*e*. count the number of flashes *etc*.), but not the goal of the experiment. Experiment 1.1 and 2.1 had seven participants (*N* = 7). Experiment 1.2, 1.3, 1.5, 2.2, 2.3, and 2.5 had the same seven participants from Experiment 1.1 and 2.1 plus one additional participant (*N* = 8). Experiments 1.4 and 2.4 had five new participants (*N* = 5). All participants reported normal or corrected-to-normal vision and normal hearing. Experiments were approved by the Caltech Committee for the Protection of Human Subjects, and all participants gave informed written consent.

### Stimuli

**Fig 2:**
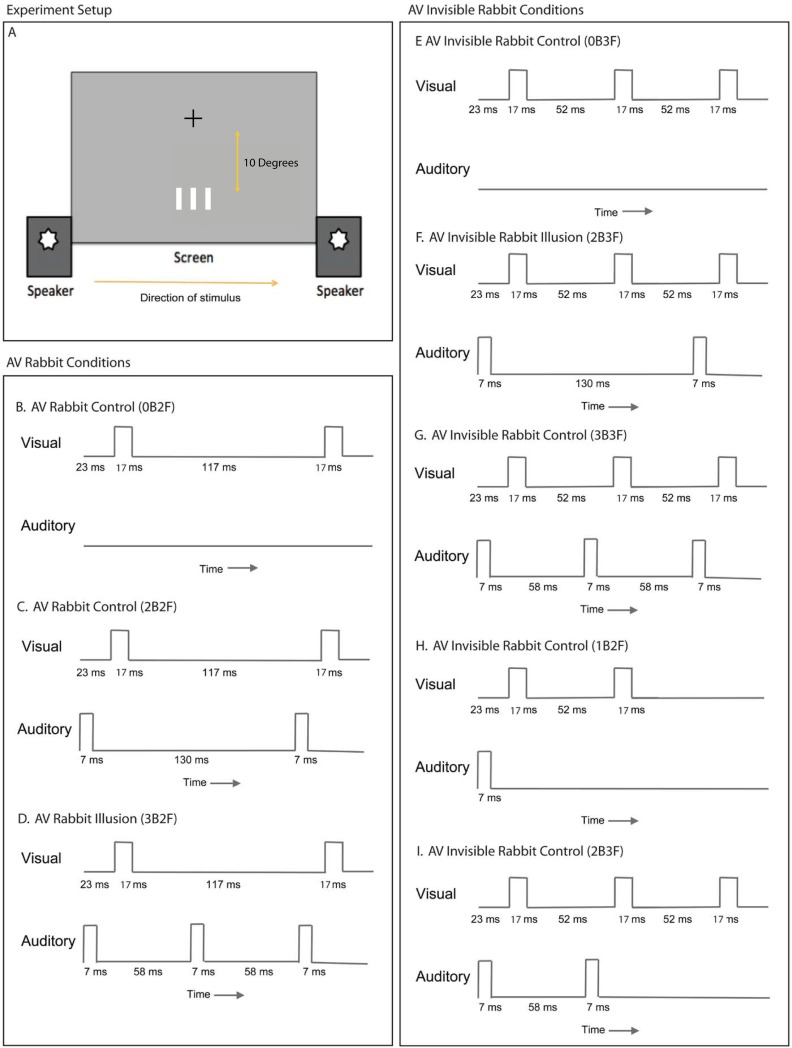
Timing and experiment setup diagrams of the illusory and invisible AV Rabbits. Diagram A shows the computer setup used to display visual flashes and generate auditory beeps. All beeps have equal loudness from the left and right speaker. Diagrams B through I indicate the relative timing of the visual and auditory stimuli in each of the experimental conditions tested in this paper.

Participants were seated 57 cm away from a monitor with a 60 Hz refresh rate (Fig 2A). The visual stimulus was a series of light gray bars (80% of the maximum screen brightness) presented on a dark gray background (30% brightness). The bars were 0.28 degrees of visual angle in width, and 1.2 degrees in length. In all experiments except the eccentricity experiment, the bars were presented 10 degrees below the fixation point (the eccentricity experiment had eccentricities at 4, 10, and 16 degrees below the fixation point). Flashes were 1.42 degrees apart when there were 3 flashes presented, and 2.84 degrees apart when two flashes were presented (this is the distance from the center of the first flash to the center of the next flash). Each flash was 17 ms in duration (one screen flip). Fig 2 shows the precise timing of each of the stimuli conditions presented. The room was dimly lit; there were no overhead lights, and ambient light was mostly shielded by a black curtain. The head was unconstrained, but was gently stabilized using a chin rest.

The auditory stimuli were presented using two speakers, which were on either side of the screen, and simultaneously presented the same auditory stimulus. Beeps were a 7 ms long 800 Hz modulated by a square wave. An oscilloscope was used to verify the correct timing of the beeps and flashes (Fig 2). We set the delay between the beeps and flashes (Fig 2) as 23 ms to be consistent with the double flash illusion as well as to account for differences in auditory and visual transmission and processing speeds [18, 19]. Our pilot observations suggested that the effects (both AV rabbit and the Invisible AV rabbit) are qualitatively and quantitatively very similar between the two delay parameters, 23 ms and 0 ms.

### Terminology

Sessions, blocks, trials, conditions, and experiments are used to identify the study parts and sections. A session was each time a participant visited the laboratory to perform the experiment, and most participants performed multiple sessions of experiments. Trials were each time a stimulus was presented and the participant answered a question about that stimulus. Blocks were each period of continuous sequential trials that are randomized in order. A majority of sessions had multiple experimental blocks that were performed with breaks between blocks as needed. Experiments were the scientific subparts of blocks, where a particular question was posed and tested. Each block could have had one to two experiments contained in each. A condition was a particular stimulus type (such as one beep and two flashes, (1b2f)) that was presented in a random order within one block.

Most participants performed several sessions (*i*.*e*. laboratory visits) to complete the required blocks. Experiment 1.3 contained trials with both left-to-right and right-to-left apparent motion, but all other experiments contained only trials with left-to-right apparent motion. Sessions that included Experiment 1.3 always started with that experiment (block 3, details below) (so as to not bias participants towards one direction of apparent motion). All other blocks were randomized in order within each session across participants, and trials within each block were presented in random order.

### Task

Participants responded using the keyboard and mouse. They reported the number of flashes they perceived using the keys 1–4 on the number pad. In some blocks, after reporting the number of flashes, participants then reported the perceived location of each flash, using the mouse to move the cursor (which was presented as a bar) in the desired location, then clicking. When reporting the flash location, the cursor started in the center of the screen (in the horizontal direction). A visual bar located in the same position as the cursor, was used to report a location, and therefore was also initially centered for location reporting. After the first location question, the presentation of the bar for the next question was where the cursor was presented at the end of the first question. For example, if the participant saw two flashes, a left flash and a right flash, the following would occur. In the first question about location of the first flash, the bar would start in the center and participant would move it to the left and click. The second question would ask about the location of the second flash, and the bar would be initially presented on the left because that is where is was presented last. Participant would then move the cursor over to the right and click. (Note: The initial cursor location was set to be at center of the screen, so if a bias were generated by initial cursor location it would likely be toward the center of the screen. Therefore, bias due to cursor position cannot explain the perceived location of the flashes in the AV rabbit between the first and last flash location.)

In other blocks, participants reported both the number of flashes they perceived and rated their confidence in their report (the options were: 1, 2, 3 and 4 where one was extremely confident and four was completely unsure). Auditory and visual stimuli were coded and responses were recorded using MATLAB 2015b and Psychophysics Toolbox 3.0.

### Stimulus types

The stimuli presented are diagrammed in Fig 2, which shows the relative timing of the auditory and visual stimuli. The number of auditory beeps and visual flashes are indicated in the text with the following format: NbMf. Where N is the number of auditory beeps and M is the number of visual flashes. In these terms, the stimuli presented included: 0b2f (Fig 2B), 0b3f (Fig 2E), 1b2f (Fig 2H), 2b2f (Fig 2C). 2b3f (Fig 2F and Fig 2I), 3b2f (Fig 2D), 3b3f (Fig 2G).

### Specific experiment blocks

#### Block 1: Experiment 1.1 and Experiment 2.1 flash counting

**Fig 3:**
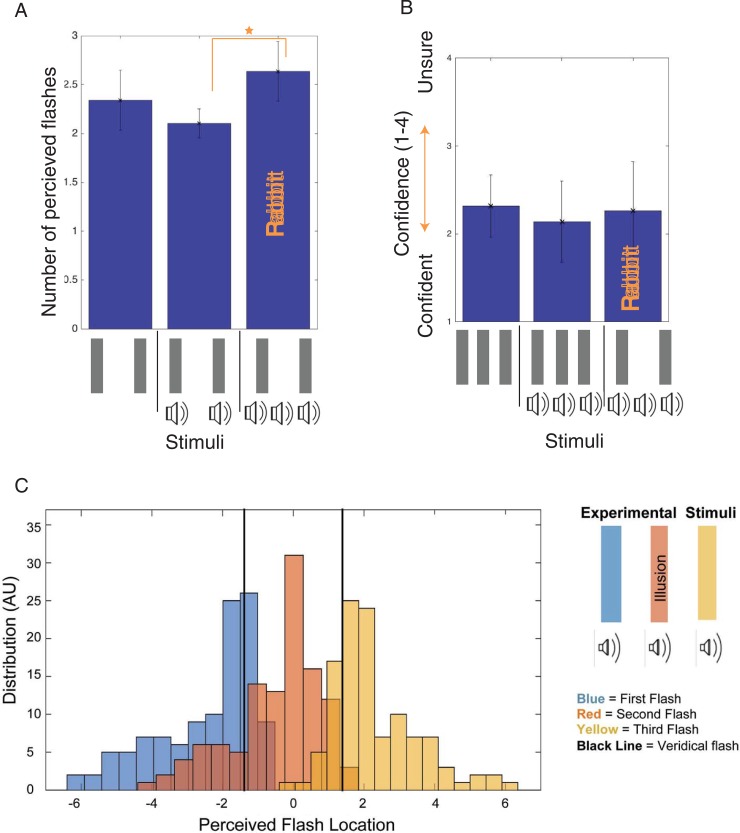
Illusory AV rabbit results. Fig 3A plots the number of flashes perceived for several beep and flash stimuli presented in Experiment 1.1. Fig 3B shows the confidence rating reported for perceiving three flashes for the Illusory AV Rabbit stimulus in comparison to non-illusory stimuli. Fig 3C plots the reported flash locations (in centimeters) across participants for the Illusory AV Rabbit stimulus, when three flashes are reported. Error bars are standard deviation.

**Fig 4:**
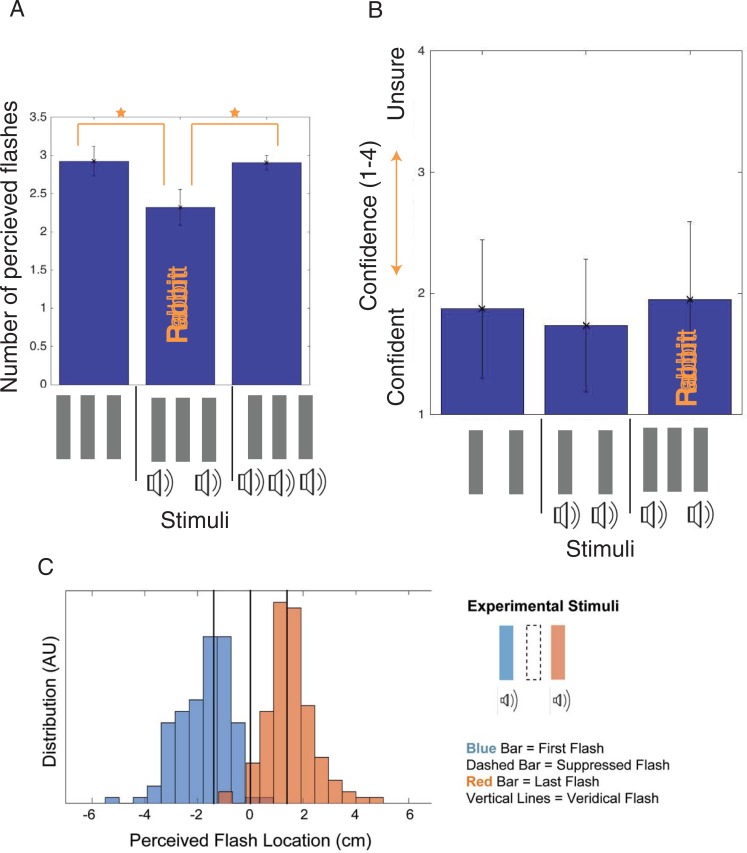
Invisible AV rabbit results. Fig 4A plots the number of flashes perceived for several beep and flash stimuli in comparison to the Invisible AV Rabbit stimulus (Experiment 2.1 and 2.2). Fig 4B shows the confidence rating reported for perceiving two flashes for the Invisible AV Rabbit stimulus in comparison to non-illusory stimuli. Fig 4C indicates the reported flash locations (in centimeters) across participants for the Invisible AV Rabbit stimulus, when two flashes are reported. Error bars are standard deviation.

The participants (*N* = 7) were presented with six conditions, consisting of (1) left flash, pause, right flash [0b2f, where 0b = 0 beeps, and 2f = 2 flashes, Fig 2B], (2) beep with left flash, pause, beep with right flash [2b2f, Fig 2C], (3) beep with left flash, beep, beep with right flash [3b2f, Fig 2D], (4) left flash, center flash, right flash [0b3f, Fig 2E], (5) beep with left flash, center flash, beep with right flash [2b3f, Fig 2F], or (6) beep with left flash, beep with center flash, beep with right flash [3b3f, Fig 2G]. Each condition was presented 25 times within the experiment block. All flashes moved left-to-right. The participants reported the number of flashes perceived (1–4) with the number pad on a keyboard. Experiment 1.1 and 2.1 aimed to provide direct evidence for the illusory rabbit (creation of an illusory visual flash by a beep) and the invisible rabbit (suppression of physically present flash by the lack of a synchronous beep).

#### Block 2: Experiment 1.2 illusory rabbit location

The participants (*N* = 8) were presented with four conditions, consisting of (1) left flash, pause, right flash [0b2f, Fig 2B], (2) beep with left flash, pause, beep with right flash [2b2f, Fig 2C], (3) beep with left flash, beep, beep with right flash [3b2f, Fig 2D], or (4) beep with left flash, beep with center flash, beep with right flash [3b3f, Fig 2G]. Each condition was randomly selected and 100 trials in total were presented during the block. All flashes moved left-to-right. The participants reported the number of flashes perceived with the number pad on a keyboard, and the location of each of the perceived (1–4) flashes by clicking with a mouse. Experiment 1.2 aimed to provide evidence for postdictive nature of illusory rabbit in terms of its location.

#### Block 3: Experiment 1.3 Prior knowledge of stimuli direction with location

The participants (*N* = 8) were presented were presented with six conditions, consisting of (1) left flash, pause, right flash [0b2f, Fig 2B], (2) beep with left flash, pause, beep with right flash [2b2f, Fig 2C], (3) beep with left flash, beep, beep with right flash [3b2f, Fig 2D], or the three conditions above with apparent motion from right to left. Each condition was presented 15 times within the experiment block. Each trial’s flashes moved either left-to-right or right-to-left; the direction was randomized across trials and the first flash was always in the same, central location. The final flash was presented at +/- 2.84 degrees. The participants reported the number of flashes perceived (1–4) with the number pad on a keyboard, and the location of each of the perceived flashes by clicking with a mouse. Experiment 1.3 aimed to further support the postdictive nature of the illusory rabbit identified in the previous experiment (1.2).

#### Block 4: Experiment 1.4 and Experiment 2.4 Eccentricity variation

The participants (*N* = 5) were presented with six conditions, consisting of (1) left flash, pause, right flash [0b2f, Fig 2B], (2) beep with left flash, pause, beep with right flash [2b2f, Fig 2C], (3) beep with left flash, beep, beep with right flash [3b2f, Fig 2D], (4) left flash, center flash, right flash [0b3f, Fig 2E], (5) beep with left flash, center flash, beep with right flash [2b3f, Fig 2F], or (6) beep with left flash, beep with center flash, beep with right flash [3b3f, Fig 2G]. Each condition was presented 25 times within the experiment block. All flashes moved left-to-right. Each condition was presented at three different eccentricities: near (4 degrees), middle (10 degrees), and far (16 degrees). The participants reported the number of flashes perceived (1–4) with the number pad on a keyboard. Experiment 1.4 and 2.4 aimed to determine if either of AV Rabbit illusions vary in strength with eccentricity from fixation.

#### Block 5: Experiment 1.5 and Experiment 2.5 reported confidence of flash perception

The participants (*N* = 8) were presented with six conditions, consisting of (1) left flash, pause, right flash [0b2f, Fig 2B], (2) beep with left flash, pause, beep with right flash [2b2f, Fig 2C], (3) beep with left flash, beep, beep with right flash [3b2f, Fig 2D], (4) left flash, center flash, right flash [0b3f, Fig 2E], (5) beep with left flash, center flash, beep with right flash [2b3f, Fig 2F], or (6) beep with left flash, beep with center flash, beep with right flash [3b3f, Fig 2G]. Each condition was presented 15 times within the experiment block. All flashes moved left-to-right. The participants reported the number of flashes perceived (1–4) with the number pad on a keyboard and the confidence of the previous response (4 options). Experiment 1.5 and 2.5 aimed to clarify the role of cognitive bias in the AV Rabbit illusions.

#### Block 6: Experiment 2.2 postdictiveness of the invisible rabbit

The participants (*N* = 8) were presented with three conditions, consisting of (1) beep with left flash, center flash [1b2f, Fig H], (2) beep with left flash, center flash, beep with right flash [2b3f, Fig 2F], or (3) beep with left flash, beep with center flash, right flash [2b3f, Fig 2I]. The condition 2 and 3 thus both contained two beeps and three flashes, but varied whether the second beep was paired with the final flash or the middle flash. Each condition was presented 25 times within the experiment block. All flashes moved left-to-right. The participants reported the number of flashes perceived (1–4) with the number pad on a keyboard. Experiment 2.2 aimed to provide evidence for the role of postdiction in the invisible rabbit illusion.

#### Block 7: Experiment 2.3 invisible rabbit location

The participants (*N* = 8) were presented with four conditions, consisting of (1) left flash, center flash, right flash [0b3f, Fig 2E], (2) beep with left flash, beep with right flash [2b2f, Fig 2C], (3) beep with left flash, center flash, beep with right flash [2b3f, Fig 2F], or (4) beep with left flash, beep with center flash, beep with right flash [3b3f, Fig 2G]. Each condition was selected randomly for a total of 100 trials within the experiment block. All flashes moved left-to-right. The participants reported the number of flashes perceived (1–4) with the number pad on a keyboard and the location of each of the perceived flashes by clicking with a mouse. Experiment 2.3 aimed to investigate the flash locations of the invisible rabbit illusion, in order to determine if the unperceived flash was either suppressed or fused with another flash.

## Results

### Statistical approach

For each participant, the mean number and the standard deviation of perceived flashes were calculated for each condition, and in many of the experiments, participants also reported perceived location of the flashes; these data were aggregated based on how many flashes participated on a given trial. In several cases, assumptions underlying parametric statistics were violated, so throughout our analyses, we used non-parametric statistics to test for differences between conditions. Initial data processing was conducted using MATLAB; statistical analyses were conducted using SPSS statistics. When recommended by SPSS (due to small number of cases), we used exact p-values based on the binomial distribution. To facilitate comparisons across experiments, we reported the standardized test statistic *z* wherever possible. Data are available via the Open Science Framework.

### Part 1: Illusory Audiovisual (AV) rabbit

#### Experiment 1.1 Illusory flashes (N = 7) (Block 1)

The first step in investigating the Illusory AV Rabbit is to show that an illusory flash is perceived due to the presence of a beep, when that beep is preceded and followed by flash-beep pairs. Therefore, in the first experiment participants recorded the number of flashes perceived when a variety of beep-flash stimuli are presented (further experimental parameters are described in the methods section). Fig 3A shows that the Illusory AV Rabbit (3b2f) stimulus caused participants to report perceiving more flashes (*Mdn* = 2.52, *M* = 2.64, *SD* = 0.31) than an identical stimulus without the second unpaired beep (2b2f) (*Mdn* = 2.04, *M* = 2.10, *SD* = 0.15). Both conditions showed significant deviations from normality using the Shapiro-Wilk test, thus we used the Related-Samples Wilcoxon Signed Rank Test to compare these conditions. This difference was statistically significant (*z = 2*.*536*, *p* = 0.011, *r* = 0.634). Therefore, the presence of an unpaired beep (preceded and followed by flash-beep pairs), is causing an illusory flash in the Illusory AV Rabbit (3b2f) stimulus. This result was replicated by Experiment 1.2, 1.3, 1.4 (different participants), and 1.5, which have different designs and goals but repeat the 3b2f (rabbit) and 2b2f conditions.

To further obtain converging statistical evidence, we conducted several additional analyses. The difference between the number of flashes perceived in the 0b2f (*Mdn* = 2.24, *M* = 2.34, *SD* = 0.31) and the 2b2f conditions was not statistically significant (*z* = 1.825, *p* = 0.068, *r* = 0.456). We also conducted one-sample Wilcoxon Signed Rank Tests to compare each of the three tested conditions to the veridical value of two flashes; in all three cases, the difference in perceived flashes was statistically significant (3b2f: *z* = 2.536, *p* = 0.011, *r* = 0.0897; 0b2f: *z* = 2.371, *p* = 0.018, *r* = 0.838; 2b2f: *z* = 2.032, *p* = 0.042, *r* = 0.718).

Finally, in order to confirm that the illusion strength was not substantially modulated by repetitive experiences of the illusion we investigated if the illusory strength significantly varied over the experimental duration (*i*.*e*. repetitions of trials). To test this, a direct comparison of the first and last 50 trials (1/3 of the trials) for the Illusory AV Rabbit (3b2f) condition was performed using the Related-Samples Wilcoxon Signed Rank Test. There was no significant difference between the first and last third of the trials for the 3b2f Illusory AV Rabbit condition (*z* = 0.676, *p* = 0.499, *r* = 0.169) or for the 2b2f condition (*z* = 0.000, *p* = 1.000, *r* = 0.000); however, there was a significant difference for the 0b2f condition (*z* = 2.383, *p* = 0.017, *r* = 0.596), such that more flashes were reported in the first 50 trials (*Mdn =* 2.35, *M* = 2.46, *SD* = 0.39) than in the last 50 trials (*Mdn =* 2.11, *M* = 2.24, *SD* = 0.32).

#### Experiment 1.2: Illusory rabbit location (N = 7–8) (Block 2)

Analysis of the reported number of flashes perceived by each participant replicated the original effect described in Experiment 1.1; participants reported seeing more flashes in the 3b2f condition (*Mdn* = 2.54, *M* = 2.54, *SD* = 0.34) than in the 2b2f (*Mdn* = 2.00, *M* = 2.05, *SD* = 0.07) or 0b2f (*Mdn* = 2.18, *M* = 2.20, *SD* = 0.19) conditions, though fewer than in the 3b3f condition (*Mdn* = 2.89, *M* = 2.81, *SD* = 0.24). We used the Related-Samples Wilcoxon Signed Rank Test to compare the 2b2f and 3b2f conditions, as these were the critical conditions for replicating our effect. The difference between the 3b2f and 2b2f conditions was statistically significant, (*z* = 2.366, *p* = 0.018, *r* = 0.592).

While the first experiment showed that an illusory flash is perceived in the Illusory AV Rabbit (3b2f), it did not indicate where that flash is located. This second experiment asks participants to report the number of flashes and then the locations of each of the flashes perceived. The trials in which three flashes were perceived for the Illusory AV Rabbit (3b2f), were used to generate a distribution of locations reported for each flash (Fig 3C). The veridical flashes were located at positions of −1.42, and 1.42 degrees.

Of the eight participants who took part in this experiment, one participant did not ever report seeing the illusory flash (this participant also reported seeing only two flashes on the majority of trials in which three physical flashes were presented), and thus only data from the other seven participants was analyzed for location. For each of the other participants, the mean reported location of each flash was calculated for each condition. When three flashes were reported, the reported positions of the first and third flashes were shifted substantially away from the center, while the second flash was perceived to be located in the approximate center of the two physical flashes. The median reported position (across observers) for the first flash was -2.40 degrees (M = –2.63 degrees, *SD* = 1.19 degrees), the median reported position for the second flash was −0.15 degrees (*M* = -0.50 degrees, *SD* = 0.97 degrees), and the mean reported position for the third flash was 2.29 degrees (*M* = 2.47 degrees, *SD* = 0.96).

We used the Jonckheere-Tempstra test, which allows testing for an ordered pattern of medians. This test does not take into account repeated measures, and thus is overly conservative for our design, but repeated measures tests such as Friedman’s ANOVA do not allow testing for a particular hypothesis about order. In this case, we hypothesized that the second flash would be perceived in between the veridical flashes. For center-to-right motion, each flash was perceived further to the right than the prior flash (*z* = 4.478, *p* < 0.001, *r* = 0.978), with all pairwise comparisons significant. Thus, we conclude that the second flash was perceived to appear at a different location from, and in between the first and third locations. This is suggestive that the Illusory AV Rabbit is postdictive in mechanism, as the third flash is not shown until after the second stimulus is finished.

We conducted several additional analyses. The first of these analyses compared the reported locations of the first and last flashes in the 3b2f condition in which an illusion was perceived with those in which an illusion was not perceived. One participant saw the illusion on every 3b2f trial, and thus was excluded from this analysis. Every one of the six remaining participants showed the same pattern of responses: reporting the first and last flash as further from the center on trials in which they experienced an illusion than trials on which they didn’t experience an illusion (see Table 1). Using the Related Samples Wilcoxon Signed Rank Test to compare the locations, we found the difference was statistically significant (*z* = 2.524, *p* = 0.031, *r* = 0.729).

**Table 1:** Experiment 1.2 illusory rabbit location.

Condition	Median, Mean, SD of Reported Location of First Flash (degrees)	Median, Mean, SD of Reported Location of Final Flash (degrees)
3b2f: three flashes reported	-2.49, -2.88, 1.07	2.61, 2.59, 0.97
3b2f: two flashes reported	-1.67, -2.19, 0.92	1.45, 1.44, 0.31

Median, mean, and SD reported locations for the flashes in the 3b2f conditions for the trials in which participants reported seeing three flashes and for the trials in which participants (*N* = 6) reported seeing two flashes. Note that the veridical locations of the first and final flash were at -1.42 and + 1.42 degrees.

Additional data analyses are included in the supplemental information.

#### Experiment 1.3: Prior knowledge of stimuli direction with location: A critical test for postdictiveness (N = 8) (Block 3)

For the number of flashes reported, the results were again consistent with that of Experiment 1.1; participants often reported seeing three flashes in the 3b2f conditions (for center-to-left motion, *Mdn* = 2.80, *M* = 2.69, *SD* = 0.34 and for center-to-right motion, *Mdn* = 2.73, *M* = 2.63, *SD* = 0.35), more frequently than in the 2b2f conditions (for center-to-left motion, *Mdn* = 2.03, *M* = 2.04, *SD* = 0.05 and for center-to-right motion, *Mdn* = 2.00, *M* = 2.05, *SD* = 0.08) and the 0b2f conditions (for center-to-left motion, *Mdn* = 2.27, *M* = 2.29, *SD* = 0.23 and for center-to-right motion, *Mdn* = 2.20, *M* = 2.27, *SD* = 0.21). Comparisons were made using the Related-Samples Wilcoxon Signed Rank Test; for both directions, significantly more flashes were perceived in the 3b2f conditions than in the 2b2f conditions (all p < 0.05).

In order to prevent prediction of the final flash location in the Illusory AV Rabbit, we randomized the direction that the flashes moved. In Experiment 1.3, participants reported the number of flashes perceived, and indicated the locations of those flashes, similar to Experiment 1.2.

Table 2 indicates the mean reported location of each flash (averaged across observers) for the trials in which participants reported seeing three flashes (Table 1). Note that the veridical locations of the first and final flash were at 0 and +/- 2.84 degrees.

**Table 2:** Experiment 1.3 Prior knowledge of stimuli direction with location.

Condition	Median, Mean, SD of Reported Location of First Flash (degrees)	Median, Mean, SD of Reported Location of Second Flash (degrees)	Median, Mean, SD of Reported Location of Final Flash (degrees)
3b2f center-to-right	-0.52, -0.58, 0.42	0.59, 0.78, 0.84	3.11, 3.21, 0.96
3b2f center-to-left	0.67, 0.57, 0.60	-0.47, -0.47, 0.90	-3.00, -3.12, 1.35

Median, mean, and SD reported locations for the flashes in the 3b2f conditions for the trials in which participants reported seeing three flashes.

Participants reported that the middle flash of the Illusory AV Rabbit (2b3f) was perceived between the first and last flash, independent of the direction of flash movement. As with Experiment 1.2, we used the Jonckheere-Tempstra test, which allows testing for an ordered pattern of medians. This test does not take into account repeated measures, and thus is overly conservative for our design, but repeated measures tests such as Friedman’s ANOVA do not allow testing for a particular hypothesis about order. In this case, we hypothesized that the second flash would be perceived in between the veridical flashes. For center-to-right motion, each flash was perceived further to the right than the prior flash (*z* = 4.867, *p* < 0.001, *r* = 0.993), with all pairwise comparisons significant. Similarly, for center-to-left motion, each flash was perceived further to the left than the prior flash (*z* = -4.444, *p* < 0.001, *r* = -0.907), with all pairwise comparisons significant. Therefore, even when the location of the final flash is unpredictable, the second illusory flash is perceived between the final and first flashes. This direction randomization further verifies that postdiction rather than prediction generates the Illusory AV Rabbit.

Additional data analyses are included in the supplemental information.

#### Experiment 1.4: Eccentricity variation of the illusory AV rabbit (N = 5) (Block 4)

It is interesting to investigate if changes to the eccentricity of the visual stimulus impact the strength of the Illusory AV Rabbit, as it does in similar illusions (such as the double flash illusion) [18, 19]. We tested this hypothesis by varying the eccentricity of the flash location between three locations (Near ~ 4 degrees, Middle ~ 10 degrees, Far ~ 16 degrees), where an increase in eccentricity may decrease the visual signal to noise ratio. Participants reported the number of flashes perceived. At all eccentricities, the Illusory AV Rabbit replicated successfully in this new group of participants. We used the Related-Samples Wilcoxon Signed Rank Test to separately test for the effect at each eccentricity by comparing the 3b2f conditions to the 2b2f conditions, and found significant differences in all three cases (all p < 0.05), confirming the replication of the illusion. We used Friedman’s ANOVA to test for differences in the effect size by examining the number of flashes perceived in the 3b2f condition perceived at each of the three locations; we found that, although there was a trend of increased illusion strength at the farthest distance (near *Mdn* = 2.86 reported flashes, *M* = 2.64 reported flashes, *SD* = 0.41; middle *Mdn* = 2.80 reported flashes, *M* = 2.79 reported flashes, *SD* = .28; far *Mdn* = 3.00 reported flashes, *M* = 2.88 reported flashes, *SD* = 0.25), the differences were not statistically significant, *X*^*2*^_*F*_(2) = 3.263, *p* = 0.196. These results indicate that the Illusory AV Rabbit could be resistant to change across eccentricity (within the tested range); if true this would draw an interesting contrast to similar illusions (such as the double flash illusion) which alter with eccentricity [18, 19]. Additional experiments with participant gaze verified by eye-tracking would further clarify the impact of eccentricity.

#### Experiment 1.5: Reported confidence of flash perception (N = 7–8) (Block 5)

It is important to test if unconscious cognitive bias (by sounds) is not causing the perception of the Illusory AV Rabbit. A confidence rating for the number of flashes perceived was used to verify that bias did not likely generate the Illusory AV Rabbit. Our argument is that if the Illusory Rabbit (in 3b2f) is due to ambiguity in the input signals and unconscious cognitive bias (by the audible sounds), then the confidence level would be much less than the control conditions with valid perception. Participants indicated how many flashes they saw and then rated their confidence in that number.

Again, the basic illusory rabbit effect replicated; we used the Related-Samples Wilcoxon Signed Rank Test and found that significantly more flashes were perceived in the 3b2f condition (*Mdn* = 2.43, *M* = 2.49, *SD* = 0.36) than in the 2b2f condition (*Mdn* = 2.00, *M* = 2.03, *SD* = 0.03), *z* = 2.201, *p* = 0.028, *r* = 0.550. We compared trials on which participants reported seeing three flashes and calculated the mean confidence rating for the 3b2f, 3b3f, and 0b3f conditions so we could compare confidence ratings for the illusory flash to confidence ratings for real flashes. One participant did not perceive the illusion, and did not report three flashes for any trials in the 3b2f condition; we excluded that participant from the confidence analyses (though not from the replication results above) and thus had a total of seven participants. Ratings of confidence were similar for the 3b2f illusory flash condition (*Mdn* = 2.33, *M* = 2.26, *SD* = 0.56, 95% CI = [1.75–2.78]) and the real flash conditions, both with beeps in the 3b3f condition (*Mdn* = 2.33, *M* = 2.14, *SD* = 0.46, 95% CI = [1.71–2.57], and when the flashes were presented without beeps in the 0b3f condition (*Mdn* = 2.29, *M* = 2.32, *SD* = 0.35, 95% CI = 1.99–2.64). We conducted a Friedman’s ANOVA and found no significant difference between the three conditions, *X*^*2*^_*F*_(2) = 1.68, *p* = 0.43. If the reported illusory flashes were due to bias, is less probable that the participants would report the same perceptual certainty for flashes they did not actually “see”. While this does not rule out the potential that the experiment is statistically underpowered, or there was cognitive misinterpretation of instructions, it does indicate that there is a similarity in confidence between the illusion and a real perceptual stimulus.

In short, the Illusory AV rabbit is, at least in part, postdictive, and the current results point to a lower-level mechanism (*i*.*e*. at a sensory-perceptual level, rather than a cognitive-level bias or construct) contributing to this effect.

### Part 2: Invisible Audiovisual (AV) rabbit

#### Experiment 2.1: Invisible rabbit flashes (N = 7) (Block 1)

The first goal with the Invisible AV Rabbit is to show that a flash is not perceived when it is preceded and followed by flash-beep pairs. Therefore, in the first Invisible AV Rabbit experiment, participants were asked to report the number of flashes perceived. As shown in Fig 4A, in the 2b3f condition, significantly fewer flashes were reported (*Mdn* = 2.36, *M* = 2.32, *SD* = 0.24) than in the 3b3f condition (*Mdn* = 2.90, *M* = 2.91, *SD* = 0.10) or the 0b3f condition (*Mdn* = 2.96, *M* = 2.93, *SD* = 0.19). We used a Related Samples Wilcoxon Signed Rank Test to compare the results of the 2b3f and 3b3f conditions to see whether the absence of a second beep suppressed perception of a flash; this difference between the 2b3f and 3b3f conditions was significant (*z* = 2.524, *p* = 0.012, *r* = 0.631). This comparison between 2b3f (Invisible AV Rabbit) and 3b3f indicates the role of the beeps in flash suppression in 2b3f and that the fewer flashes perceived with 2b3f was not merely due to difficulty with quick flash perception in the visual modality. Instead, the flash suppression is due to a crossmodal effect resulting from the absence of a beep with the second flash. This result was replicated by Experiment 2.3, 2.4 (different participants), and 2.5, which have different designs and goals but repeat the 2b3f (invisible rabbit) and 3b3f conditions.

We conducted several additional analyses to probe this effect. Using a Related Samples Wilcoxon Signed Rank Test, we found that the difference between the 2b3f and 0b3f conditions was significant (*z* = 2.524, *p* = 0.012, *r* = 0.631). We also compared the median response for each of the three conditions to the veridical value of three flashes using a One-Sample Wilcoxon Signed Rank Test. While the median perceived number of flashes in the 0b3f condition did not significantly differ from 3 flashes (*z* = -0.775, *p* = 0.438, *r* = -0.274), it did significantly differ from 3 flashes in both the 3b3f condition (*z* = -2.060, *p* = 0.039, *r* = -0.728 and the 2b3f condition (*z* = -2.527, *p* = 0.012, *r* = -0.894).

Finally, in order to confirm that the illusion strength was not substantially modulated by repetitive experiences of the illusion we investigated if the illusory strength significantly varied over the experimental duration (*i*.*e*. repetitions of trials). To test this, a direct comparison of the first and last 50 trials (1/3 of the trials) for the Invisible AV Rabbit (2b3f) condition was performed using the Related-Samples Wilcoxon Signed Rank Test. There was no significant difference between the first and last third of the trials for the 2b3f Invisible AV Rabbit condition (*z* = 0.314, *p* = 0.753, *r* = 0.079), the 3b3f condition (*z* = 0.000, *p* = 1.000, *r* = 0, or the 0b3f condition (*z* = -0.105, *p* = 0.917, *r* = 0.026).

#### Experiment 2.2: Postdictiveness of the invisible rabbit (N = 8) (Block 6)

Experiment 2.1 indicated that the second flash of the Invisible AV Rabbit is not perceived. Yet is still unclear if this is caused by the flash-beep pair before or the flash-beep after the flash in question. If the flash-beep pair following the suppressed flash influences the suppression, then the effect is likely postdictive in mechanism. To test this, participants indicated the number of flashes perceived for a series of flash-beep configurations. Using the Related-Samples Wilcoxon Signed Rank Test, it was found that participants perceived significantly fewer flashes. (*z* = 2.207, *p* = 0.027, *r* = 0.552), for the Invisible AV Rabbit (2b3f, Fig 2F, *Mdn* = 2.20 *M* = 2.25, *SD* = 0.20) than for [beep-(left flash), beep-(center flash), right flash] (2b3f, Fig 2I, *Mdn* = 2.48, M = 2.45, *SD* = 0.35), *t*(7) = 2.73, *p* = 0.0294, *d* = .96. Therefore, the lone flash is suppressed the most if the beep-flash pairs are before and after the lone flash, rather than just before. This indicates that postdictive processing significantly contributes to the suppression of the lone flash in the Invisible AV Rabbit. In the 1b2f condition, participants perceived fewer than 2 flashes (*Mdn* = 1.62, *M* = 1.64. *SD* = .33); a One-Sample Wilcoxon Signed Rank Test found that this was significantly different from the veridical value of 2 flashes (*z* = 2.207, *p* = 0.027, *r* = 0.780).

Prediction, in addition to postdiction, seems to contribute to suppression of the lone flash. Comparing perception in the 0b3f condition (M = 2.93 flashes, measured in Experiment 2.1) to the number of flashes reported for the 2b3f condition where the lone flash is last (M = 2.45 flashes), shows that adding beeps to the first two flashes diminishes the perception of flashes. Therefore, the difference between these two conditions indicates that there is a predictive suppression of the third flash due to the multimodal pairing of the first two beep-flash pairs. However, the predictive element to the stimulus processing does not obviate postdiction from also playing a role. The comparison between the 2b3f condition where the lone flash is last (*M* = 2.45 flashes) to the 2b3f condition when the lone flash is second (*M* = 2.25 flashes) is to this point. This comparison shows a further significant decrement in the number of flashes perceived beyond the decrement generated by prediction. Therefore, postdiction and prediction both contribute to the Invisible AV Rabbit.

#### Experiment 2.3: Invisible rabbit location (N = 8) (Block 7)

Results of the previous Experiment (2.2) show that the beep-flash pair must follow the lone flash for the strongest Invisible AV Rabbit effect. However, two potential mechanisms could cause this reduction in flash perception: the suppression of a flash, or the fusion of two flashes. In this experiment participants are asked to report the location of the two flashes they perceive with the Invisible AV Rabbit Illusion (2b3f, Fig 2F). If suppression is occurring, then the two reported flash locations should be centered on the veridical location of the first and last real flashes (second flash suppressed). If fusion is occurring, then the first or second perceived flash should be centered *between* the veridical location of the first and second or second and third real flashes.

This dataset successfully replicated the invisible rabbit effect. We used the Related-Samples Wilcoxon Signed Rank Test and found that participants reported seeing significantly fewer flashes when separately compared to the 2b3f condition (*Mdn* = 2.11, *M* = 2.19, *SD* = 0.23) than in the 3b3f condition (Mdn = 2.88, *M* = 2.88, *SD* = 0.11), and to the 0b3f condition (*Mdn* = 2.71, *M* = 2.73, *SD* = 0.030); for both comparisons, *z* = 2.521, *p* = 0.012, *r* = 0.630. In the 2b2f condition, participants almost always reported seeing two flashes (*Mdn* = 2.02, *M* = 2.04, *SD* = 0.06).

We analyzed the reported positions of the flashes for the 2b3f trials in which participants reported seeing two flashes. As shown in Fig 4C, participants perceived an initial flash close to the location of the first veridical flash, and perceived the subsequent flash close to the location of the third veridical flash. The veridical flashes were located at positions of −1.42, 0, and 1.42 degrees. One-sample Wilcoxon Signed Rank Tests were used to compare the locations of the reported flashes to the veridical locations of the flashes. The median reported position for the first flash was -1.52 degrees (*M* = −1.83, *SD* = 0.82), which was not significantly different from −1.42 degrees (*z* = -0.980, *p* = 0.327, *r* = 0.346), but was significantly different from 0 degrees (*z* = -2.521, *p* = 0.012, *r* = -0.891). The median reported position for the second flash was 1.57 degrees (*M* = 1.42, SD = 0.61), which was significantly different from 0 degrees (*z* = 2.521, *p* = 0.012, *r* = 0.891) and not from 1.42 degrees (*z* = 0.700, *p* = 0.484, *r* = 0.247). In other words, the lack of beep paired with the second veridical flash suppresses its perception, with its location information also suppressed or lost (rather than merged into the first or the third location).

#### Experiment 2.4: Eccentricity variation of the invisible rabbit (N = 5) (Block 4)

The sensitivity of the Invisible AV Rabbit to the eccentricity of the visual stimulus relative to fixation was investigated. In particular, it was expected that the illusion may be stronger at farther eccentricities, where vision has a lower spatial resolution which may allow audition to dominate vision more readily (as in the double flash illusion). We tested this hypothesis by varying the eccentricity of the flash location between three locations (Near ~ 4 degrees, Middle ~ 10 degrees, Far ~ 16 degrees), where an increase in eccentricity decreases the visual signal-to-noise ratio. At all eccentricities, the Invisible AV Rabbit replicated successfully in this new group of participants. We used the Related-Samples Wilcoxon Signed Rank Test to separately test for the effect at each eccentricity by comparing the 2b3f conditions to the 3b3f conditions, and found significant differences in all three cases (all p < 0.05), confirming the replication of the illusion. We used Friedman’s ANOVA to test for differences in the effect size by examining the number of flashes perceived in the 2b3f condition. We found that, consistent with experiment 1.4, there was a trend of increased illusion strength at the farthest distance (near *Mdn* = 2.18 reported flashes, *M* = 2.32 reported flashes, *SD* = 0.39; middle *Mdn* = 2.18 reported flashes, *M* = 2.20 reported flashes, *SD* = 0.23; far *Mdn* = 2.05 reported flashes, *M* = 2.13 reported flashes, *SD* = 0.16), but this difference was not statistically significant, *X*^*2*^_*F*_(2) = 5.158, *p* = 0.076. The results indicate that the Invisible AV Rabbit could be robust across eccentricity, drawing an interesting contrast to similar illusions (such as the double-flash illusion) which have a significant alteration with eccentricity [18, 19].

#### Experiment 2.5: Reported confidence of flash perception (N = 8) (Block 5)

A confidence rating experiment was used next to indicate if unconscious bias generated the perception of the Invisible AV Rabbit. Participants indicated how many flashes they saw and then rated their confidence in that number.

Again, the basic invisible rabbit effect replicated; we used the Related-Samples Wilcoxon Signed Rank Test, and found that significantly fewer flashes were perceived in the 2b3f condition (*Mdn* = 2.73, *M* = 2.24, *SD* = 0.29) than in the 3b3f condition (*Mdn* = 2.90, *M* = 2.89, *SD* = 0.12), (z = 2.524, p = 0.012, r = 0.631). We compared participants’ confidence that they perceived two flashes in the invisible 2b3f condition (*Mdn* = 1.93, *M* = 1.95, *SD* = 0.64, 95% CI = [1.41–2.48]) to the confidence they had for the perception of two real flashes in the 0b2f (*Mdn* = 1.81, *M* = 1.87, *SD* = 0.57, 95% CI = [1.40–2.35]) and 2b2f conditions (*Mdn* = 1.69, *M* = 1.74, *SD* = 0.55, 95% CI = [1.28–2.20])(Fig 4B). We conducted a Friedman’s ANOVA comparing the confidence ratings for trials in which participants reported seeing two flashes for the three different conditions. There was no significant difference in confidence ratings between the perception of an absent flash and a suppressed flash (*X*^*2*^_*F*_(2) = 1.00, *p* = 0.607). If the Invisible AV Rabbit were due to bias, is improbable that the participants would report the same confidence for a biased response that is not perceptual, as a real perceptual stimulus. Therefore, similarity in confidence between the illusion and a real perceptual stimulus indicates that the illusion is likely perceptual as well.

## Discussion

### Illusory AV rabbit

The Illusory AV Rabbit effect demonstrated the creation of a new illusory visual percept by a particular sequence of auditory-visual events. In particular, a spatial shift of an illusory flash was induced by a flash-beep pair following it (Fig 1A shows the Illusory AV Rabbit stimulus sequence: flash-beep, beep, flash-beep).

In the Illusory AV Rabbit effect an illusory flash is generated at the time of the second beep (shown in Experiment 1.1). The illusory flash is perceived second in the perceptual sequence and the location of the illusory flash is perceived mid-way between the first and last flash (shown in Experiment 1.2). When the direction of the flash movement is randomized between left-to-right and right-to-left, thus no prior knowledge on the direction of apparent motion was available, the illusory flash is still perceived centered between the first and last flash (Experiment 1.3). This randomization result confirms that postdiction (instead of prediction based on prior knowledge on the direction of motion) generates the spatial shift in the illusory flash toward the last flash. If the distance between the flashes and fixation point (*i*.*e*. eccentricity of the stimulus set) is varied, the illusory flash in the Illusory AV Rabbit is still perceived (even at approximately 4 degrees from fixation) indicating a potential robustness relative to signal-to-noise changes between the peripheral and central visual perception (Experiment 1.4). In other words, the visual perception may not be required to be in the periphery (and therefore noisy) in order for audition to alter it in the Illusory AV Rabbit. Finally, the Illusory AV Rabbit was shown to be likely a perceptual effect, as opposed to a cognitive effect, judging from the result of the confidence rating experiment, where the illusory flash perception was perceived with the same confidence as a real flash (Experiment 1.5).

The experiments for the Illusory AV Rabbit indicate that an illusory flash is positioned postdictively between a previous and future flash location. It indicates that audition can modify the location of a visual percept via postdictive processing.

### Invisible AV rabbit

The Invisible AV Rabbit occurs when a real flash is suppressed by the presence of a beep-flash pair following it (Fig 1B). The Invisible AV Rabbit stimulus sequence starts with a flash-beep, then a flash, and another flash-beep, where the second flash is not perceived (Experiment 2.1), and suppression of the second flash is caused by the presence of the last flash-beep pair. (Experiment 2.2). Furthermore, the last flash and second flash are not fused, as when the flash locations are reported the last flash location is centered at actual last flash location, and not between the second and last flash (Experiment 2.3). If the distance from the flash location to the fixation point (*i*.*e*. eccentricity of the stimulus set) is varied, the Invisible AV Rabbit seems to occur at up to approximately 4 degrees from fixation, indicating that even with a stronger visual signal (and less noise), audition can dominate over vision (Experiment 2.4). Finally, the Invisible AV Rabbit was found to be perceptual, as opposed to cognitive or inferential, by having similar confidence rating between the Invisible AV Rabbit perception, and real flash perception (Experiment 2.5).

The experiments for the Invisible AV Rabbit indicate that a real flash is suppressed by the presence of a beep-flash pair preceding and following it. It indicates that audition can postdictively suppress visual perception.

### Models of postdictive processing

Models of postdictive brain processes include proposed computational encoding mechanisms [16], neural mechanisms of information flow [1], and neural processing implications to visual awareness [15].

The computational approach was used by Goldreich and Tong (2013), to model the joint role of postdiction and prediction in perceptual effects by using a Bayesian low-speed prior. This model was used successfully to explain the cutaneous rabbit illusion [16], by assuming that the brain expects objects to move slowly (low-speed prior). In particular, two tactile taps on the forearm in separate locations are perceived to move a shorter distance than reality (a “length contraction”) due to the noisy position perception and the assumption the object moved slowly. Further experiments will have to be performed to test the applicability of Goldreich’s model to the AV Rabbit. Additional AV Rabbit experiments with variation in the second beep onset, would detail the relationship between the second beep timing and the reported illusory flash location. In particular, these types of experiments (with variation in the second beep onset) would indicate if the flash position is “contracted” in its position relative to the beep timing.

Shimojo (2014) described several neuroscientific models, where the information from a later stimulus could be integrated with a previous stimulus within the sensory processing pathway. In addition to the “catch-up” model detailed in the introduction of this paper, Shimojo proposed several other unimodal postdictive mechanisms, including differential processing speeds in different neural layers and/or back-projection of “fast” signals from a higher layer to a lower layer within one modality. These types of models based on brain architecture and pathways can be easily adapted to a multisensory stimulus. Models for *multimodal* postdiction include differential neural pathway processing speeds between visual and auditory processing (a modified “catch-up model”), feedback of previous stimuli information from multisensory regions to primary sensory regions and then feedforward of the integrated stimuli (feedback-feedforward model), and slower integration times in multisensory regions relative to the primary sensory regions (a different variation of the “catch-up” model). Multisensory postdiction increases the likely number of brain regions involved in postdictive processes, incorporating multisensory regions as well as multiple primary sensory regions (such as audition or vision). As such, the incorporation of multisensory postdiction both increases the types of models possible, and also makes them more specific to crossmodal integration (rather than possibly occurring in any single sensory system).

Finally, the visual awareness explanation or model [15] argues that perceptual postdiction originates from the observation that visual awareness does not occur in real time but rather is delayed and typically requires re-entry or feedback. This allows for an event up to about 80 ms following a stimulus to affect the perception of that stimulus. This type of perceptual processing model is also implicitly assumed within the neuroscientific models detailed above. Certainly, the results in this paper indirectly support this concept as an underlying but necessary principle for multisensory postdictive processing.

### Relation to multisensory integration and crossmodal interactions

Predictive models and illusions have largely dominated multisensory interactions; they include effects such as speeded audio-visual cuing, temporal ventriloquism, the McGurk Effect auditory-visual language illusion, and Maximum Likelihood Estimation (MLE) in multisensory integration [20–27]. The brain seems to be using prior information stored about each modality, as well as direct attention, to integrate multimodal cues and effectively predict future stimuli. The AV rabbit is not fully explained by these predictive models of audiovisual interaction. In particular, our control experiments indicate that the sensory information presented before the illusory flash is not sufficient to move the illusory flash toward the final flash location. However, the necessity of postdiction to explain the illusory flash location, does not mean that temporal ventriloquism, sensory uncertainty, and MLE do not contribute to generating the illusory flash, binding the auditory-visual stimuli, and generating the perceived audiovisual timing. In effect, the AV rabbit is an excellent example of a multimodal illusion that relies both on predictive processing, as well postdictive processing within the sensory regions of the brain (as indicated in experiments 1.2, 1.3, and 2.2). Our main claim is that in addition to predictive processing, postdiction is also necessary to fully explain this illusion.

Neuroscience research has begun to investigate the emerging area of multimodal postdiction, where later stimuli can affect prior stimuli. In fact, a study showed that the flash-lag effect, which is modeled as postdictive (at least in some theories), can occur crossmodally, or audio-visually [10]. The AV Rabbit Illusions add to this growing body of postdictive effects bridging the senses, and point to a new mechanism for sensory combination within short perceptual time scales. As the number of instances of crossmodal postdiction increases, the neural processing underlying postdictive interactions may become more clear, and thereby broaden the types of processing within the sensory regions of the brain. Furthermore, such crossmodal cases may further bridge the gap between lower-level, unimodal sensory postdiction and higher-level, long-term, cognitive postdiction [1, 26]. In addition, one may speculate that such postdictive processes are universal across various time-scales and various parts/spatial scales of the brain, and they evolved due to a biological advantage of saving memory and yet having predictive power for the future.

The AV Rabbit illusions also highlight an effect where audition dominates over visual perception. Auditory dominance over vision is relatively unusual; the majority of crossmodal illusions in the normally sighted show that vision modulates audition, especially spatially (such as the ventriloquist effect and McGurk effect [26]). The Colavita effect is an example of visual dominance over audition [28]. In this effect, participants are asked to respond as quickly as possible to indicate whether a flash or beep stimulus was presented. Oddball trials, explained to the participants as equipment malfunctions, presented a flash and beep simultaneously. Participants typically respond to the bimodal stimulus with predominantly the visual flash response button. The reason that the Colavita effect has visual dominance and the AV Rabbit illusions do not, may rest on the duration of the visual flash, which is significantly longer in the Colavita stimulus (typically over 150 ms, as the stimulus was presented until the participant response was registered) than the duration of the flash in the AV Rabbit illusions (20 ms). The longer flash in the Colavita stimulus likely increases the reliability of vision relative audition, and may lead to more visual dominance [22]. The short duration of the flash in the AV Rabbit illusions makes the visual percept shorter, noisier, and therefore less likely to be dominant. A very short visual stimulus duration (or alternatively high visual frequency) is a unifying principle for illusions with auditory dominance over vision, with the double flash illusion [18–19], and Shipley’s flicker-flutter effect [27] both using this domain. In addition, the Colavita effect is performed as a dual task, where the participant has to respond to a bimodal stimulus as a flash (one button press) or a beep (a different button). This type of competition between two types of tasks does not occur in our AV rabbit illusions, in which participants are instructed to ignore the number of beeps presented [29]. Finally, the AV Rabbit illusion contains a sequence of beeps and flashes, in which the stimuli before and after the illusory stimulus are influential to the illusion perceived. The Colavita effect has only one beep and/or flash without a sequence of stimuli.

### Relation to the double flash illusion

The AV Rabbit Illusions in many ways build upon the Double Flash Illusion discovered by Shams and colleagues [18–19]. The Double Flash Illusion indicated that a quick flash can be doubled by pairing it with two short beeps. The AV Rabbit uses the initial double flash paradigm to generate the second illusory flash, but further influences the location of that illusory flash via a second real shifted flash. Furthermore, the Invisible AV Rabbit indicates that the opposite of the Double Flash can occur; a visual flash can be suppressed by audition, rather than generated. These new AV Rabbit Illusions show that the auditory-to-visual interaction is not just an excitatory connection between the senses, but rather the auditory influence on vision can also cause suppression, and spatial displacement. This type of fine-tuned interaction portrays a multisensory integration that is specific to the type of information conveyed across the senses. Multisensory cortical regions may play a key role in this fine-tuned cross-sensory processing, and could be directly influencing visual cortex through feedback connections [30–33].

## Supporting information

S1 Movie A demonstration of the illusory AV rabbit. This movie plays an example demonstration of the Illusory AV Rabbit. For comparison, it also shows the visual stimuli of the Illusory AV Rabbit without sound. For the best perception make the video fill the full computer screen and turn up the audio of your computer to the maximum setting.
(MP4)

S2 Movie A demonstration of the invisible AV rabbit. This movie plays an example demonstration of the Invisible AV Rabbit. For comparison, it also shows the visual stimuli of the Invisible AV Rabbit without sound. For the best perception make the video fill the full computer screen and turn up the audio of your computer to the maximum setting.
(MP4)

S1 Table Experiment 1.2 illusory rabbit location. Median, mean, and SD reported locations for the flashes in the 3b2f condition for the trials in which participants (*N* = 7) reported seeing three flashes and for the trials 3b3f condition for the trials in which participants reported seeing three flashes. Note that the actual locations of the first and final flash were at -1.42 and + 1.42 degrees.
(DOCX)
