# Correction: What you saw is what you will hear: Two new illusions with audiovisual postdictive effects

**DOI:** 10.1371/journal.pone.0207894

**Published:** 2018-11-20

**Authors:** The PLOS ONE Staff

Figs 3 and 4 are incorrect. The orange labels on the bar graphs are incorrectly blurred. The labels should read: Rabbit. The publisher apologizes for the errors. The authors have provided corrected versions here.

**Fig 3:**
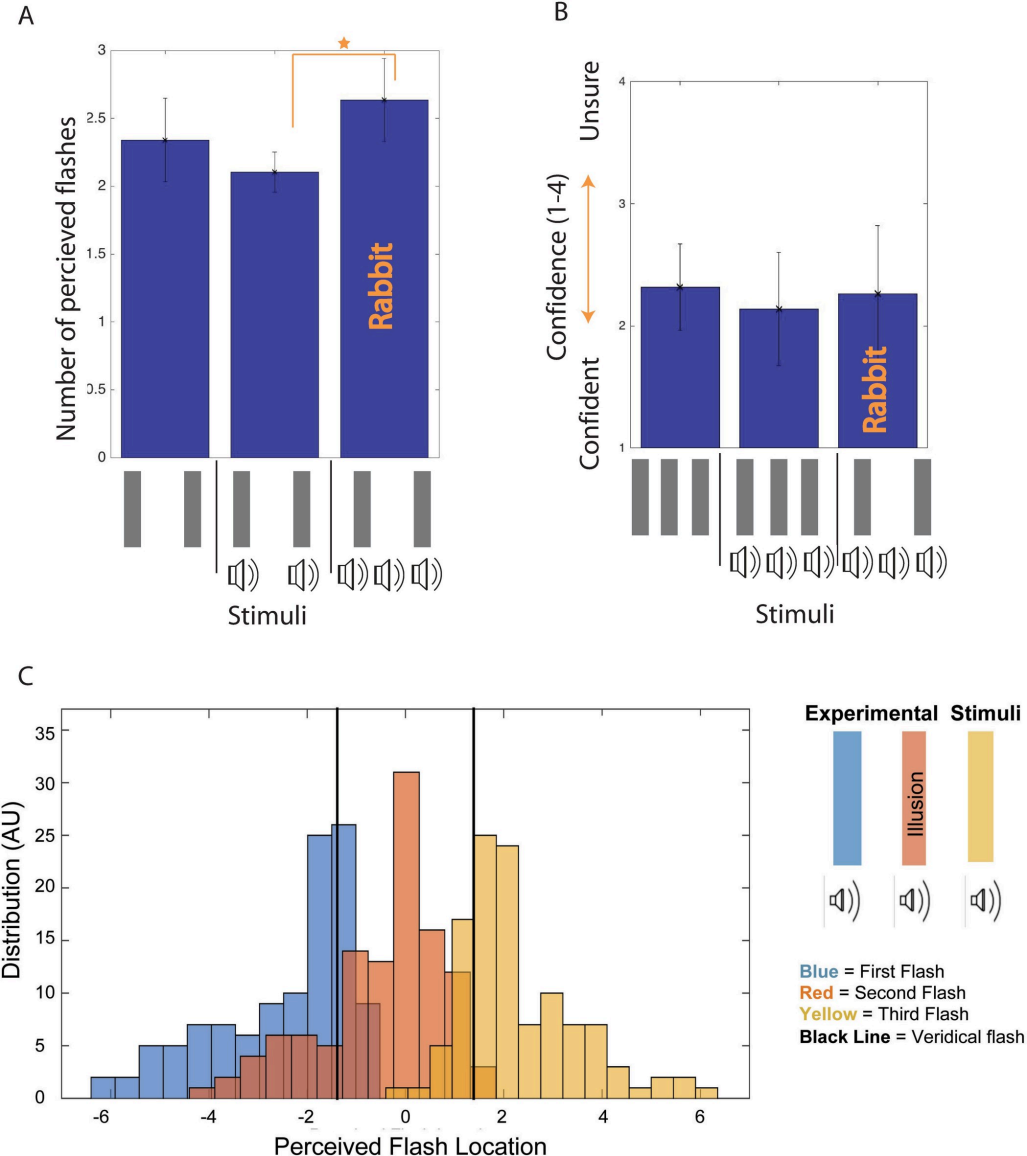
Illusory AV rabbit results. Fig 3A plots the number of flashes perceived for several beep and flash stimuli presented in Experiment 1.1. Fig 3B shows the confidence rating reported for perceiving three flashes for the Illusory AV Rabbit stimulus in comparison to non-illusory stimuli. Fig 3C plots the reported flash locations (in centimeters) across participants for the Illusory AV Rabbit stimulus, when three flashes are reported. Error bars are standard deviation.

**Fig 4:**
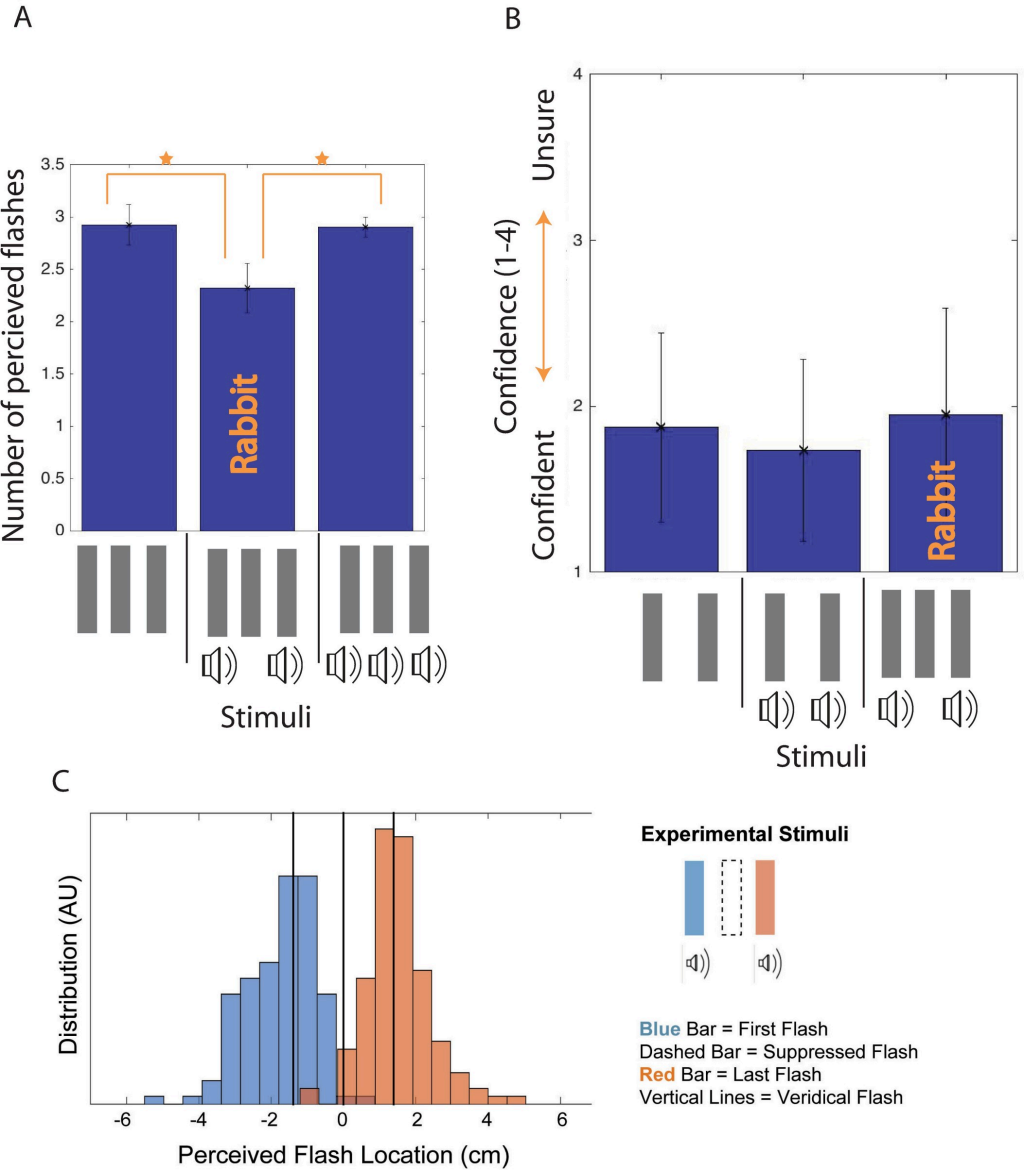
Invisible AV rabbit results. Fig 4A plots the number of flashes perceived for several beep and flash stimuli in comparison to the Invisible AV Rabbit stimulus (Experiment 2.1 and 2.2). Fig 4B shows the confidence rating reported for perceiving two flashes for the Invisible AV Rabbit stimulus in comparison to non-illusory stimuli. Fig 4C indicates the reported flash locations (in centimeters) across participants for the Invisible AV Rabbit stimulus, when two flashes are reported. Error bars are standard deviation.
