# Supplementary material for: What you saw is what you will hear: Two new illusions with audiovisual postdictive effects
Source: PLoS One. 2018 Oct 3;13(10):e0204217. doi: 10.1371/journal.pone.0204217 (PMC6169875; doi:10.1371/journal.pone.0204217)
Supplement: S1 Table — Median, mean, and SD reported locations for the flashes in the 3b2f condition for the trials in which participants (N = 7) reported seeing three flashes and for the trials 3b3f condition for the trials in which participants reported seeing three flashes. Note that the actual locations of the first and final flash were at -1.42 and + 1.42 degrees. (DOCX) [file pone.0204217.s003.docx]

**Supplemental Information**

**Additional Data and Results**

**Part 1: Illusory Audiovisual (AV) Rabbit**

**Experiment 1.2: Illusory Rabbit location (N = 7-8) (Block 2)**

We further probed the tendency for the first and last flashes to be perceived as “pushed” away from the center by comparing the reported locations of the flashes on the 3b2f trials in which illusions were perceived to the reported locations of the flashes on the 3b3f trials in which three flashes were reported. Table S1 gives the reported locations of each of the flashes; using the Related Samples Wilcoxon Signed Rank Test to compare the locations, we found the difference in reported location for the first flash was statistically significant (*z* = 2.268, *p* = 0.016, *r* = 0.606), but not for the second (*z* = 1.152, *p* = 0.125, *r* = 0.308), or final flash (*z* = -0.756, *p* = 0.453, *r* = -0.202). These statistical distributions of the first and the last flash are not itself our focus of interest, but provide the framework to assess the perceived location of the illusory second flash.

**S1 Table. Experiment 1.2 Illusory Rabbit Location.**

| Condition | Median, Mean, SD of Reported Location of First Flash (degrees) | Median, Mean, SD of Reported Location of Second Flash (degrees) | Median, Mean, SD of Reported Location of Final Flash (degrees) |
| --- | --- | --- | --- |
| 3b2f: three flashes reported | -2.40, -2.62, 1.19 | -0.15, -0.50, 0.97 | 2.29, 2.47, 0.96 |
| 3b3f: three flashes reported | -1.78, -2.25, 1.15 | -0.18, -0.28, 0.61 | 2.05, 2.17, 0.55 |

**S1 Table. Experiment 1.2 Illusory Rabbit Location.** Median, mean, and SD reported locations for the flashes in the 3b2f condition for the trials in which participants (*N* = 7) reported seeing three flashes and for the trials 3b3f condition for the trials in which participants reported seeing three flashes. Note that the actual locations of the first and final flash were at -1.42 and + 1.42 degrees.

We also compared the precision of the location reporting of the flashes. We operationalized precision by calculating each participant’s standard deviation of reported locations for each of the three flashes reported on in the 3b2f trials in which three flashes were reported as well as the precision of the locations in the 3b3f trials in which three flashes were reported. The precision of the first, second, and final flashes in the 3b2f and 3b3f the middle illusory flash did not significantly differ, and the precision of the illusory middle flash in the 3b2f condition did not differ from the precision of the first and final flashes in that 3b2f conditions (Related Samples Wilcoxon Signed Rank Test, all *p* > 0.05).

**Experiment 1.3: Prior knowledge of stimuli direction with location: A critical test for postdictiveness (N = 8) (Block 3)**

To further follow up the mixed finding in Experiment 1.2 that the illusory flash “pushes” out the perceived location of the veridical flashes, we also compared the reported positions of the first and last flashes in the 3b2f conditions for the trials in which an illusion was perceived to the reported positions of the first and last flashes when an illusion was not perceived. For all four comparisons (left and right motion for the first and the last flash), there was no significant difference in reported flash location (Related Samples Wilcoxon Signed Rank Test, all *p* > 0.05).
